# Symmetric and asymmetric dimethylarginine as risk markers of cardiovascular disease, all-cause mortality and deterioration in kidney function in persons with type 2 diabetes and microalbuminuria

**DOI:** 10.1186/s12933-017-0569-8

**Published:** 2017-07-11

**Authors:** Emilie H. Zobel, Bernt Johan von Scholten, Henrik Reinhard, Frederik Persson, Tom Teerlink, Tine W. Hansen, Hans-Henrik Parving, Peter K. Jacobsen, Peter Rossing

**Affiliations:** 1 Steno Diabetes Center Copenhagen, Niels Steensens Vej 2, 2820 Gentofte, Denmark; 2 Department of Clinical Chemistry, VU University Medical Center, Amsterdam, The Netherlands; 3 Department of Endocrinology, Rigshospitalet, University of Copenhagen, Copenhagen, Denmark; 4 The Heart Center, Rigshospitalet, University of Copenhagen, Copenhagen, Denmark; 5 Department of Clinical Medicine, University of Copenhagen, Copenhagen, Denmark

**Keywords:** Microalbuminuria, Type 2 diabetes, Cardiovascular disease, Macrovascular disease, Symmetric dimethylarginine, Asymmetric dimethylarginine

## Abstract

**Background:**

To evaluate symmetric dimethylarginine (SDMA) and asymmetric dimethylarginine (ADMA) as risk markers of cardiovascular disease, all-cause mortality and deterioration in renal function in a well characterised type 2 diabetic population with microalbuminuria and without symptoms of coronary artery disease.

**Methods:**

200 participants followed for 6.1 years. SDMA and ADMA were measured at baseline. Endpoints included (1) composite cardiovascular endpoint (n = 40); (2) all-cause mortality (n = 26); and (3) decline in eGFR of >30% (n = 42). Cox models were unadjusted and adjusted for traditional risk factors (sex, age, systolic blood pressure, LDL-cholesterol, smoking, HbA_1c_, creatinine and urinary albumin excretion rate). To assess if SDMA or ADMA improved risk prediction beyond traditional risk factors we calculated c statistics and relative integrated discrimination improvement (rIDI). C statistic (area under the curve) quantifies the model’s improved ability to discriminate events from non-events. rIDI quantifies the increase in separation of events and non-events on a relative scale.

**Results:**

Higher SDMA was associated with increased risk of all three endpoints (unadjusted: p ≤ 0.001; adjusted: p ≤ 0.02). Higher ADMA was associated with all-cause mortality (unadjusted: p = 0.002; adjusted: p = 0.006), but not cardiovascular disease or decline in eGFR (p ≥ 0.29).The c statistic was not significant for any of the endpoints for either SDMA or ADMA (p ≥ 0.10). The rIDI for SDMA was 15.0% (p = 0.081) for the cardiovascular endpoint, 52.5% (p = 0.025) for all-cause mortality and 48.8% (p = 0.007) for decline in eGFR; for ADMA the rIDI was 49.1% (p = 0.017) for all-cause mortality.

**Conclusion:**

In persons with type 2 diabetes and microalbuminuria higher SDMA was associated with incident cardiovascular disease, all-cause mortality and deterioration in renal function. Higher ADMA was associated with all-cause mortality. SDMA and ADMA significantly improved risk prediction for all-cause mortality, and SDMA for deterioration in renal function beyond traditional risk factors.

**Electronic supplementary material:**

The online version of this article (doi:10.1186/s12933-017-0569-8) contains supplementary material, which is available to authorized users.

## Background

Cardiovascular disease is a major complication in type 2 diabetes despite multifactorial intervention [1, 2]. There is an ongoing search for biomarkers that can improve risk prediction in type 2 diabetes. Symmetric and asymmetric dimethylarginine (SDMA and ADMA, respectively) are dimethylarginines structurally related to l-arginine. Due to their biological functions, both markers have been explored as cardiovascular biomarkers. ADMA is considered an independent risk factor for cardiovascular disease and mortality in a range of populations at different levels of cardiovascular risk [3, 4] and in type 1 diabetes [5, 6]. Although elevated levels of ADMA in persons with type 2 diabetes and macrovascular disease have been reported in cross sectional studies [7, 8], there are conflicting results concerning the prognostic value [9, 10]. It is unknown whether associations seen for ADMA extend to the structural isomer SDMA. A recent meta-analysis demonstrated that higher SDMA is a risk factor for cardiovascular disease and mortality in different populations, with the strongest associations observed in the general population [3]. This meta-analysis did not report results for any diabetic cohorts. Studies of SDMA in type 2 diabetes are few; however a cross sectional study demonstrated higher SDMA in persons with cardiovascular disease [8].

The use of metabolomics has been instrumental in identifying new biomarkers of chronic kidney disease such as the dimethylarginines [11]. ADMA and SDMA were originally reported to accumulate in renal failure [12] and may also be risk factors for deterioration in renal function in type 2 diabetes. ADMA is an outcome predictor of acute kidney injurie [13] and has been reported to predict an increased rate of decline in GFR and development of end stage renal disease in type 1 diabetes [5] and has been related to presence of renal dysfunction in type 2 diabetes [8, 14].

We evaluated SDMA and ADMA as risk markers of cardiovascular disease, all-cause mortality and decline in renal function in a well characterised type 2 diabetic population with microalbuminuria and without symptoms suggestive of coronary artery disease. Moreover, we assessed whether SDMA and ADMA improved risk prediction beyond traditional risk factors using c statistics and integrated discrimination improvement (IDI).

## Methods

### Participants

200 participants were recruited from the outpatient clinic at Steno Diabetes Center Copenhagen. Inclusion criteria were (1) type 2 diabetes according to WHO criteria; (2) no history of coronary artery disease or symptoms suggestive of cardiac disease (assessed from interviews with the patient and patient records); and (3) persistent urinary albumin excretion rate (UAER) >30 mg/24 h (in two out of three consecutive measurements). 613 consecutive patients were invited by letter to participate in the study. 72 patients declined the invitation. 341 patients were excluded. Exclusion criteria were (1) normal or non-persistent elevated UAER (n = 52); (2) symptoms/signs or a history of cardiac disease, including Q waves in 12-lead ECGs (n = 180); (3) relative contraindications to computed tomography angiography or coronary angiography, including abnormal plasma creatinine levels (n = 86); (4) physical or mental disability (n = 10); or (5) malignancy (n = 13) [15].

At the time the study was designed, in the absence of data from other prospective studies using the biomarkers in a type 2 diabetic population, the study was conducted as an exploratory study using the sample size available.

### Biochemical and other measures

Plasma concentrations of SDMA and ADMA were determined simultaneously by high-performance liquid chromatography with fluorescence detection as previously described [16], using modified chromatographic separation conditions [17]. For both SDMA and ADMA, the intra- and interassay coefficients of variation were <2 and <4%, respectively. Blood was centrifuged immediately after collection, and plasma was frozen at −80 °C and stored in a research biobank for analysis immediately after the last participant was examined. Thus the maximal storage time of the samples prior to analysis of both biomarkers was 13 months. Quantification of SDMA and ADMA was available for all participants. UAER was measured in 24-h collected urine samples by enzyme immunoassay (Vitros, Raritan, NJ, USA). Current smoking was defined as one or more cigarettes, cigars or pipes per day. Brachial blood pressure was measured twice after 10 min rest using an appropriate cuff size, and averaged.

### Follow up

All participants were traced through the Danish National Death Register and the Danish National Health Register on the 1st of January 2014. No participants were lost to follow-up. Definitions of the three predefined endpoints have previously been described [18, 19]. The combined cardiovascular endpoint was defined as cardiovascular mortality, stroke, ischaemic cardiovascular disease and heart failure. For participants with multiple events, only the first was included. Moreover, we followed 177 out of the 200 (88.5%) participants originally included with yearly measurements of plasma creatinine used for calculations of eGFR applying the Chronic Kidney Disease Epidemiology Collaboration (CKD-epi) equation [20]. The renal endpoint was defined as decline in eGFR >30% (evaluated as change from baseline to the last available measurement), as proposed by Coresh et al. [21].

### Statistical analyses

Symmetric dimethylarginine, ADMA and UAER were non-normally distributed and are summarized as median with interquartile range (IQR), and log10 transformed in all analyses. All other continuous variables are given as mean ± standard deviation (SD) and categorical variables as total numbers with corresponding percentages.

We used *t*-test for continuous variables and Χ^2^ test for categorical variables to test for differences in potential confounders in the population categorized according to SDMA and ADMA above or below the median.

First, we applied Cox proportional hazards analysis to compute the unadjusted hazard ratios (HR)’s per 1 SD increment of SDMA and ADMA with 95% confidence interval (CI) for the three endpoints, next we calculated HR’s adjusted for traditional cardiovascular risk factors based on existing evidence: sex, age, systolic blood pressure, LDL-cholesterol, smoking, HbA_1c_, plasma creatinine and UAER.

To quantify the added predictive value of SDMA and ADMA, we calculated receiver operating characteristic (ROC) curves based on logistic regression models and applied c statistics to compare the area under the curve (AUC) for the model including traditional cardiovascular risk factors and the AUC for the model including traditional cardiovascular risk factors plus SDMA and ADMA, respectively. For the significant associations in the adjusted models, we further illustrated the risk information with ROC-curves. We then calculated the IDI, a measure suggested by Pencina et al. [22] as a more powerful method to demonstrate improved diagnostic performance of a biomarker. To ease the interpretation, relative IDI (rIDI) is provided and reported as a percentage. rIDI is defined as the increase in discrimination slope when adding SDMA or ADMA respectively to traditional risk factors divided by the slope of the model including only the traditional risk factors [22].

Finally, we applied the Kaplan–Meier failure function to compare the risks of the combined cardiovascular endpoint, all-cause mortality and deterioration in renal function according to the median level of SDMA, and to compare the risks of all-cause mortality according to the median level of ADMA.

A two-tailed *p* value of <0.05 was considered significant. Statistical analyses were performed using SPSS for Windows (version 23.0, Chicago, IL, USA) and SAS software (version 9.4, SAS Institute, Cary, NC, USA).

## Results

### Patient characteristics

**Fig. 1:**
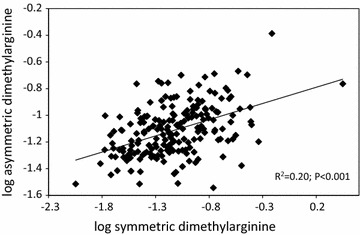
Correlation between symmetric and asymmetric dimethylarginine

Baseline characteristics of all the 200 participants and categorized according to the median level of SDMA and ADMA respectively are listed in Table 1. Participants with high SDMA were older, had higher plasma creatinine, lower eGFR and systolic blood pressure compared to participants with low SDMA. Participants with high ADMA levels were older, with higher UAER and plasma creatinine and lower eGFR compared to participants with low ADMA. All participants were receiving multifactorial treatment on top of oral antidiabetic medication or insulin including antihypertensive drugs (99%), renin–angiotensin–aldosterone system-blocking treatment (98%), statins (95%) and aspirin (92%). Figure 1 illustrates the positive correlation between SDMA and ADMA (R^2^ = 0.20; p < 0.001).

**Table 1:** Clinical characteristics of the study population at baseline categorized according to SDMA and ADMA values below or above the median

Characteristics	All participants (n = 200)	Symmetric dimethylarginine		p value	Asymmetric dimethylarginine		p value
		<0.4525 µmol/l (n = 100)	≥0.4525 µmol/l (n = 100)		<0.4625 µmol/l (n = 100)	≥0.4625 µmol/l (n = 100)	
Male, *n* (%)	*152 (76)*	76 (76)	75 (75)	0.87	77 (23)	74 (74)	0.62
Age (years)	*59* *±* *9*	56.4 ± 9.2	60.9 ± 7.6	*<0.001*	57.2 ± 9.8	60.1 ± 7.3	*0.015*
Known duration of diabetes (years)	*13* *±* *7*	10.8 ± 6.6	14.8 ± 7.6	*<0.001*	11.7 ± 7.3	13.9 ± 7.3	*0.033*
Body mass index (kg/m^2^)	*32.6* *±* *5.8*	33.0 ± 5.3	32.1 ± 6.2	0.24	32.3 ± 4.8	32.8 ± 6.6	0.48
HbA_1c_ (%)	*7.9* *±* *1.3*	7.93 ± 1.36	7.80 ± 1.34	0.49	7.92 ± 1.41	7.81 ± 1.29	0.55
HbA_1c_ (mmol/mol)	*63* *±* *14*	63 ± 14.9	62 ± 14.6	0.49	63 ± 15.4	62 ± 14.1	0.55
Urinary albumin excretion rate (mg/24-h)	*103 (39–230)*	104.5 (47.9–219.8)	98.0 (38.0–241.0)	0.86	80.5 (33.0–176.9)	133.0 (56.0–303.0)	*0.011*
P-creatinine (μmol/L)	*76* *±* *18*	67.0 ± 13.8	85.9 ± 17.4	*<0.001*	72.0 ± 17.4	80.9 ± 18.2	*<0.001*
eGFR (ml/min/1.73 m^2^)	*130* *±* *17*	99.3 ± 13.0	79.7 ± 15.6	*<0.001*	94.6 ± 16.9	84.4 ± 16.3	*<0.001*
Systolic blood pressure (mmHg)	*152* *±* *76*	133 ± 18	127 ± 16	*0.012*	130 ± 17	130 ± 17	0.60
Diastolic blood pressure (mmHg)	*75* *±* *11*	77 ± 11	73 ± 11	*0.016*	76 ± 11	73 ± 11	*0.051*
LDL cholesterol (mmol/L)	*1.85* *±* *0.78*	1.90 ± 0.88	1.81 ± 0.67	0.39	1.81 ± 0.78	1.90 ± 0.78	0.45
Current smoker, *n* (%)	*59 (30)*	34 (34)	25 (25)	0.16	31 (31)	28 (28)	0.64
Treatment with							
Oral antidiabetic, *n* (%)	*170 (85)*	87 (87)	83 (83)	0.43	89 (89)	81 (81)	0.11
Insulin, *n* (%)	*124 (62)*	58 (58)	66 (66)	0.24	59 (59)	65 (65)	0.38
Antihypertensive drugs, *n* (%)	*198 (99)*	100 (100)	98 (98)	0.16	99 (99)	99 (99)	1.00
RAAS blockade, *n* (%)	*196 (98)*	96 (96)	100 (100)	*0.043*	98 (98)	98 (98)	1.00
Beta-blocker, *n* (%)	*27 (14)*	9 (9)	18 (18)	*0.063*	11 (11)	16 (16)	0.31
Calcium channel blockers, *n* (%)	*80 (40)*	43 (43)	37 (37)	0.39	36 (36)	44 (44)	0.25
Diuretics, *n* (%)	*128 (64)*	57 (57)	41 (41)	*0.039*	63 (63)	65 (65)	0.77
Statin, *n* (%)	*189 (95)*	94 (94)	95 (95)	0.76	97 (97)	92 (92)	0.12
Aspirin, *n* (%)	*183 (92)*	92 (92)	91 (91)	0.80	90 (90)	93 (93)	0.45

p values for differences between participants with symmetric and asymmetric dimethylarginine below or above the median

Italic values indicate significance of p value (p < 0.05)

*eGFR* estimated glomerular filtration rate, *RAAS* renin-angiotensin-aldosterone system

### Incidence of cardiovascular disease, all-cause mortality and decline in eGFR

Median (IQR) follow up for the combined cardiovascular endpoint and mortality was 6.1 (5.9–6.6) years.

#### Combined cardiovascular endpoint (n = 40)

11 fatal cardiovascular events (two cases of acute myocardial infarction, one case of ischaemic cardiovascular disease, six sudden and otherwise unexplained deaths and two cases of heart failure) and 29 non-fatal cardiovascular events (three cases of acute myocardial infarction, three strokes, 19 cases of ischaemic cardiovascular disease, four cases of heart failure all leading to hospital admission).

#### All-cause mortality (n = 26)

11 related to cardiovascular disease, 9 cancer-related and 6 related to other causes.

#### Decline in eGFR >30% (n = 42)

Median (IQR) follow up was 4.9 (3.8–5.4) years. In 23 participants the decline was confirmed in two or more measurements. In 17 participants the decline was seen at the final study visit and could therefore not be confirmed. No participants progressed to dialysis or end-stage renal disease during follow up.

The distribution of time to event for the combined cardiovascular endpoint, all-cause mortality and deterioration in renal function is shown in Additional file 1: Figure S1.

### Symmetric dimethylarginine as a risk marker

Table 2 shows the association between SDMA and cardiovascular disease, all-cause mortality, and decline in eGFR. Higher SDMA was associated with all endpoints in both unadjusted (p ≤ 0.001) and adjusted (p ≤ 0.02) analyses.

**Table 2:** Biomarkers in relation to risk of fatal and nonfatal cardiovascular events, all-cause mortality and decline in eGFR >30%

Biomarker	Model	Cardiovascular events (n = 40)	p	All-cause mortality (n = 26)	p	Decline in eGFR > 30% (n = 42)	p
Symmetric dimethylarginine log scale (1 SD = 0.06)	Unadjusted	1.5 (1.2–1.9)	*0.001*	1.6 (1.2–2.1)	*0.001*	1.9 (1.3–2.6)	*<0.001*
	Adjusted	1.7 (1.1–2.6)	*0.019*	2.3 (1.4–3.9)	*0.001*	2.2 (1.4–3.7)	*0.002*
Asymmetric dimethylarginine log scale (1 SD = 0.13)	Unadjusted	1.2 (0.8–1.6)	0.38	1.7 (1.2–2.5)	*0.002*	1.2 (0.8–1.6)	0.29
	Adjusted	1.0 (0.7–1.5)	0.93	1.8 (1.2–2.7)	*0.006*	1.0 (0.7–1.4)	0.85

Values are hazard ratios with 95% confidence intervals, and represent 1 SD increment of log-transformed values of the biomarkers. Adjustment included sex, age, systolic blood pressure, LDL cholesterol, smoking, HbA_1c_, plasma creatinine, and urinary albumin excretion rate

Italic values indicate significance of p value (p < 0.05)

*eGFR* estimated glomerular filtration rate

**Fig. 2:**
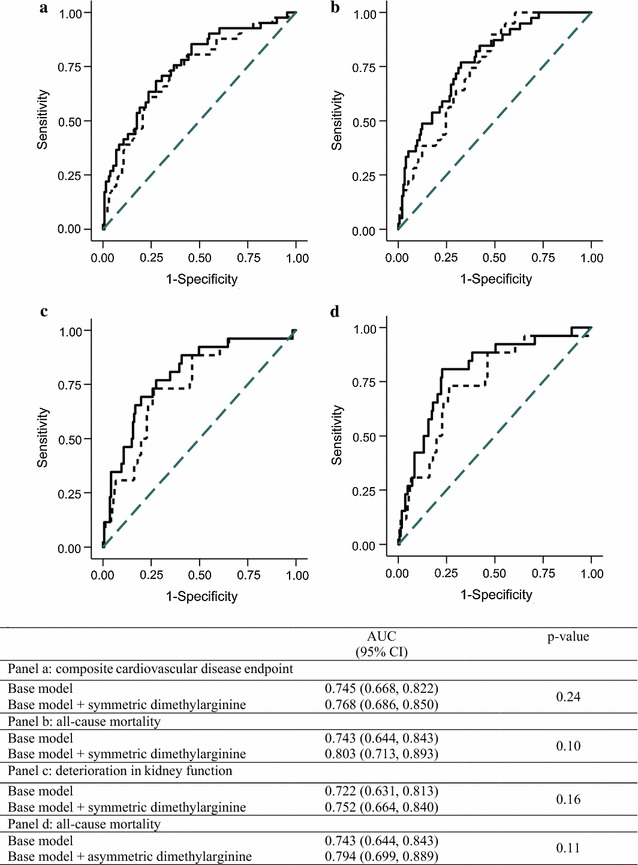
Receiver operating characteristic (ROC) curves. Base model includes sex, age, systolic blood pressure, LDL-cholesterol, smoking, HbA_1c_, creatinine and urinary albumin excretion rate. *Dashed line* reference; *Dotted line* Base model; *Full line* Base model + symmetric dimethylarginine/asymmetric dimethylarginine

As shown in Fig. 2a, b in relation to the composite cardiovascular endpoint, the AUC increased from 0.745 (95% CI 0.668–0.822) to 0.768 (0.686–0.850), and in relation to all-cause mortality, the AUC increased from 0.743 (0.644–0.843) to 0.803 (0.713–0.893) after adding SDMA to the model including traditional risk factors. As shown in Fig. 2c for decline in eGFR >30% the AUC increased from 0.722 (0.631–0.813) to 0.752 (0.664–0.840). Increase in AUC (c statistic) quantifies the model’s improved ability to discriminate events from non-events. In simpler terms, if the model including SDMA on top of traditional risk factors is more likely to assign higher risk to persons with events. These changes in the AUC were all non-significant (p ≥ 0.10). Pencina et al. [22] have described how AUC does not change materially even for powerful predictors and the increase in AUC is directly affected by the robustness of the baseline model. IDI statistics have been suggested by Pencina to further evaluate the incremental contribution of a new biomarker. rIDI quantifies the increase in separation of events and non-events on a relative scale. The rIDI was 15.0% (p = 0.081) for the cardiovascular endpoint, 52.5% (p = 0.025) for all-cause mortality and 48.8% (p = 0.007) for decline in eGFR >30%.

The cumulative incidence of decline in eGFR was higher in participants with SDMA level above the median (p = 0.0003; Additional file 1: Figure S2 panel c). However, the cumulative incidence of cardiovascular disease and all-cause mortality was similar in participants with SDMA level above or below the median (p ≥ 0.098; Additional file 1: Figure S2 panel a and b).

### Asymmetric dimethylarginine as a risk marker

Table 2 shows the association between ADMA and cardiovascular disease, all-cause mortality, and decline in eGFR. In unadjusted analysis, higher ADMA was associated with all-cause mortality (p = 0.002), but not with cardiovascular disease (p = 0.38) or decline in eGFR >30% (p = 0.29). The association between ADMA and all-cause mortality persisted after adjustment for traditional risk factors (p = 0.006). As shown in Fig. 2d when adding ADMA to traditional risk factors the AUC increased from 0.743 (95% CI 0.644–0.843) to 0.794 (0.699–0.889) in relation to all-cause mortality. The change was non-significant (p = 0.11). The rIDI for ADMA was 49.1% (p = 0.017) for all-cause mortality.

The cumulative incidence of all-cause mortality was similar in participants with ADMA level above or below the median (p = 0.74; Additional file 1: Figure S2 panel d).

## Discussion

In our cohort of 200 persons with type 2 diabetes and microalbuminuria, we demonstrated higher SDMA to be an independent determinant of incident cardiovascular disease, all-cause mortality and decline in renal function. We further demonstrated higher ADMA to be associated with all-cause mortality after adjustment for traditional risk factors, while ADMA was not associated with incident cardiovascular disease and decline in renal function. When applying c statistics, SDMA and ADMA respectively added to traditional risk factors did not significantly increase the risk prediction. IDI statistics has been suggested by Pencina as a necessary and more powerful method going beyond statistical significance and c statistic to evaluate the incremental contribution of a new biomarker [22]. We demonstrated added predictive value using IDI statistics for all-cause mortality after addition of SDMA and ADMA, respectively; and for decline in renal function adding SDMA to traditional risk factors.

Symmetric dimethylarginine is without direct inhibitory effect on nitric oxide synthesis and has therefore been given little attention compared to ADMA [12]. We are only aware of two studies that have investigated the association of SDMA to incident cardiovascular disease in persons with type 2 diabetes. In 270 persons with type 2 diabetes Hsu et al. [23] reported elevated SDMA to predict risk of cardiovascular events (cardiovascular death, non-fatal myocardial infarction and stroke) in univariate analysis, but significance was lost after adjustment. Median follow-up was 5.7 years. A recent study by Anderssohn et al. reported an association of SDMA with prevalent cardiovascular disease in 783 older persons with type 2 diabetes. This association lost significance after adjusting for age, sex and renal function. After 4 years of follow-up SDMA was not significantly associated with risk of incident coronary artery or cerebrovascular disease [9]. In our cohort we showed an association between higher SDMA and incident cardiovascular disease, all-cause mortality as well as deterioration in kidney function. SDMA has emerged as a marker of renal disease and in contrast to ADMA which is cleared mainly through enzymatic action [24], SDMA is almost completely excreted by the kidneys [25]. Levels of SDMA have previously been shown to be closely related to glomerular filtration rate in a cross-sectional study in persons with coronary artery disease [26]. This relation was confirmed in the present study, where high levels of SDMA were associated with lower levels of eGFR at baseline. Importantly, in our cohort SDMA was associated with deterioration in kidney function after adjustment for kidney function at baseline. Besides the strong correlation with renal function, the underlying mechanism of SDMA as a marker of risk in diabetes could potentially be explained by a pro-inflammatory effect. Where ADMA directly inhibits the production of nitric oxide by interfering with the nitric oxide synthase, SDMA acts as a competitor of l-arginine, the substrate for nitric oxide synthase. This ultimately leads to an increased endothelial production of reactive oxygen species [26, 27] and this pro-inflammatory effect of SDMA may trigger vascular pathology. We demonstrated a moderate correlation between SDMA and ADMA. However, the relationship between SDMA and ADMA may not be straightforward and they may reflect different aspects of pathophysiology. We suggest SDMA as a promising new marker of endothelial dysfunction and inflammation with the potential to improve risk prediction in persons with type 2 diabetes and microalbuminuria.

The lack of association between ADMA and incident cardiovascular disease is in contrast to a number of previous studies. However, the prospective studies of ADMA in type 2 diabetes have mainly included subjects at high cardiovascular risk including manifest cardiovascular disease [23, 28–30]. Krzyzanowska et al. demonstrated an independent association between high ADMA and manifest cardiovascular disease (myocardial infarction, percutaneous coronary intervention, coronary-artery bypass graft, stroke, carotid revascularization and all-cause mortality) in 125 participants with type 2 diabetes after a median follow-up of 21 months [tertile III vs tertile I: HR 2.37 (95% CI 1.05–5.35), p = 0.038] [29]. Of the 125 participants, 40% had a history of macrovascular disease. Hsu et al. reported elevated ADMA to predict risk of cardiovascular events when analyzed both as categorical [tertile III vs tertile I: HR 2.3 (95% CI 1.1–4.8), p = 0.026] and as continuous variable [per 1 SD (0.09 µmol/l) increase HR: 1.30 (95% CI 1.01–1.68), p = 0.04] after appropriate adjustment [23]. 78% of the participants had concomitant coronary artery disease at baseline. Cavusoglu et al. reported elevated levels of ADMA to be independently associated with an increased risk of cardiovascular outcomes at 2-years of follow-up in high-risk type 2 diabetic males with known or suspected coronary artery disease referred for coronary angiography [tertile III vs tertile I–II: composite outcome of all-cause mortality, myocardial infarction or stroke HR 2.00 (1.10–3.62), p = 0.02] [30]. In contrast, two larger studies, the Framingham Offspring study enrolling 3320 participants (372 with diabetes) and a study in 997 individuals (359 with diabetes) referred for coronary angiography demonstrated higher ADMA to be associated with cardiovascular events and all-cause mortality only in the participants without diabetes at baseline [10, 31]. In the study including 783 older persons with type 2 diabetes, the association between higher ADMA and prevalent cardiovascular disease lost significance after adjustment for age, sex and renal function, and in the prospective analysis ADMA was not associated with incident coronary artery or cerebrovascular disease [9].

There is some evidence that both glycaemic control and nephropathy status may directly affect ADMA levels [23, 32, 33]. Therefore, ADMA levels may change with progression of disease and complications status and the prognostic value of ADMA in persons with diabetes could therefore be highly dependent on the study population investigated. This could explain the conflicting results concerning the prognostic value of ADMA for cardiovascular complications in type 2 diabetes. Our study population differs from previous studies as we excluded participants with a history of or symptoms suggestive of coronary artery disease. In contrast we did find a relation to all-cause mortality. This is in accordance with findings in the aforementioned study by Cavusoglu et al. [tertile III vs tertile I–II: all-cause mortality HR 2.63 (1.13–6.11), p = 0.03] [30]. Other authors have included all-cause mortality in a combined cardiovascular endpoint and reported positive associations [29].

The ratio of SDMA/ADMA has been suggested as a predictive biomarker for decline in renal function in persons with type 2 diabetes [14]. In our study the SDMA/ADMA ratio was associated with all-cause mortality (p = 0.039), but not with cardiovascular disease or deterioration in kidney function (p ≥ 0.12) in analysis adjusted for traditional risk factors.

### Clinical implications

We demonstrate that the addition of SDMA to traditional risk factors improved risk prediction for all-cause mortality and decline in renal function and our findings illustrate that SDMA may be useful in the risk assessment of persons with type 2 diabetes. However, evidence suggests that the role of both SDMA and ADMA in diabetes is complex and not completely understood. Therefore, further research is needed in type 2 diabetic subjects at different risk of cardiovascular and renal disease to further elucidate the role of the two dimethylarginines.

### Study limitations

The main limitation is the relatively small sample size. Moreover, it is important to consider that we included persons with type 2 diabetes and microalbuminuria recruited from a single center, limiting the generalisation of our results. For both SDMA and ADMA we report non-significant improvements in risk prediction evaluated with c statistics. However, we demonstrated added predictive value using IDI statistics. It remains a challenge to establish the ranges of meaningful improvement for IDI and the clinical value of ADMA and SDMA as risk markers remains to be evaluated in interventions studies.

## Conclusion

In a well-characterized population with type 2 diabetes, microalbuminuria and without a history or symptoms suggestive of coronary artery disease, higher SDMA was associated with incident cardiovascular disease, all-cause mortality and deterioration in renal function, and improved risk prediction for all-cause mortality and renal decline beyond traditional risk factors. Higher ADMA improved risk prediction for all-cause mortality beyond traditional risk factors.

## Additional file

**Additional file 1: Figure S1.** Distribution of time to event for the combined cardiovascular endpoint, all-cause mortality and deterioration in renal function. **Figure S2.** Kaplan–Meier failure function estimates.

## Abbreviations

ADMA: asymmetric dimethylarginine
AUC: area under the curve
eGFR: estimated glomerular filtration rate
IDI: integrated discrimination improvement
rIDI: relative integrated discrimination improvement
ROC: receiver operating characteristic
SDMA: symmetric dimethylarginine
UAER: urinary albumin excretion rate
