# Supplementary material for: Symmetric and asymmetric dimethylarginine as risk markers of cardiovascular disease, all-cause mortality and deterioration in kidney function in persons with type 2 diabetes and microalbuminuria
Source: Cardiovasc Diabetol. 2017 Jul 11;16:88. doi: 10.1186/s12933-017-0569-8 (PMC5505150; doi:10.1186/s12933-017-0569-8)
Supplement: Supplementary file 1 — Additional file 1: Figure S1. Distribution of time to event for the combined cardiovascular endpoint, all-cause mortality and deterioration in renal function. Figure S2. Kaplan–Meier failure function estimates. [file 12933_2017_569_MOESM1_ESM.docx]

**Figure S1.** Distribution of time to event for the combined cardiovascular endpoint, all-cause mortality and deterioration in renal function

**Figure S2.** Kaplan–Meier failure function estimates

Kaplan–Meier failure function estimates for (a) the composite cardiovascular endpoint b) all-cause mortality c) deterioration in renal function and in two categories of the levels of SDMA (above or below the median level, 0.4525 µmol/l); and (d) all-cause mortality in two categories of the levels of ADMA (above or below the median level, 0.4625 µmol/l).

a)

b)

c)

d)

Numbers refer to participants in each category at risk at the beginning of each 2 year interval. Dashed line, above median SDMA/ADMA; solid line, below median SDMA/ADMA.
